# Children’s migration and chronic illness among older parents ‘left behind’ in China

**DOI:** 10.1016/j.ssmph.2017.10.002

**Published:** 2017-10-08

**Authors:** Maria Evandrou, Jane Falkingham, Min Qin, Athina Vlachantoni

**Affiliations:** Centre for Research on Ageing and ESRC Centre for Population Change, University of Southampton, Southampton SO17 1BJ, UK

**Keywords:** Migration, Chronic illness, Older parents, Left behind, China

## Abstract

The relationship between adult children’s migration and the health of their older parents ‘left behind’ is an emerging research area and existing studies reflect mixed findings. This study aims to investigate the association between having migrant (adult) children and older parents’ chronic illness in China, using chronic stomach or other digestive diseases as a proxy. Secondary analysis of the national baseline survey of the 2011 China Health and Retirement Longitudinal Study (CHARLS) was conducted. Analyses were conducted in a total of sample of 6495 individuals aged 60 years and above from 28 out of 31 provinces in China, who had at least one child at the baseline survey. Binary logistic regression was used. The prevalence of any of the diagnosed conditions of chronic stomach or other digestive diseases was higher among older people with a migrant son than among those without (27 percent vs 21 percent, p < 0.001). More specifically, the odds ratio of reporting a disease was higher among older adults with at least one adult son living in another county or province than among those with all their sons living closer (OR = 1.29, 95% CI = 1.10–1.51). The results from this large sample of older adults support the hypothesis that migration of sons significantly increases the risk of chronic stomach and other digestive diseases among ‘left behind’ elderly parents in contemporary China.

## Highlights

• Older adults with at least one migrant son were more likely to report a chronic stomach or other digestive disease than others.
• Being female was independently associated with a higher risk of reporting such diseases.
• Rural vs. urban, and regional, disparity were independently associated with the risk of reporting the chronic digestive disorders.

## 1 Introduction

China has the largest aged population in the world today and the pace of population ageing is much faster than in many other high-income or low- and middle-income countries (Du, 2013). Following this demographic shift is a health transition from maternal, child and communicable disorders to chronic non-communicable diseases (World Health Organization (WHO), 2015). The increasing levels of non-communicable diseases are influenced by a small set of common and modifiable risk factors such as an unhealthy diet, physical inactivity and tobacco use; frequently exacerbated by the adverse impact of rapid and unplanned urbanization on individuals’ health profile through a greater exposure to shared risk factors (WHO, 2005).

Notwithstanding the debate on the extent to which the parents of migrant children are ‘left behind’ (Biao, 2007, Knodel et al., 2010), the relationship between adult children’s migration and the health of their older parents ‘left behind’ is an emerging research area and existing studies reflect mixed findings. The positive effects of children’s out-migration on their parents’ health in the origin community have been evidenced in Thailand and Indonesia, in part reflecting the economic gains of migration through remittances (Abas et al., 2009, Kuhn et al., 2011), negative effects have been found in India and Mexico (Falkingham et al., 2017, Antman, 2010).

In the Chinese context, the family has been the major source of support and care for elderly members, and filial piety - a fundamental belief in Chinese culture - has emphasized a son's duty to respect and support his parents (Chou, 2011). Support from adult children has been shown to have effects in counteracting stress among older people resulting from inequities experienced in the public domain, for instance in terms of medical care and pension protection, both in urban and rural areas (Sun, 2004, Cong and Silverstein, 2008). However, traditional systems of family support from adult children in later life are now coming under pressure as a result of mass internal migration; in 2015, there were an estimated 277.5 million rural labourers working in China’s cities, constituting 36 percent of China's total workforce of around 770 million (National Bureau of Statistics of the People’s Republic of China, 2017). In the absence of a mature system of old age social protection, including pensions and health and social care, it is posited that the out-migration of an adult son may lead to his parents’ deteriorating emotional and physical care, affecting their health in the long run. This paper aims to add to the literature in this field by investigating the association between adult children’s migration and older parents’ chronic illness (interchangeable with chronic disease) in China using a recently available national representative sample. In a previous paper, the authors found a significant association between having a migrant son and their older parents reporting a lifestyle-related chronic disease measured as diagnosed hypertension, diabetes and heart disease in India (Falkingham et al., 2017). In order to explore the health consequences of globalization and urbanization in greater detail, this study applies diagnosed conditions of stomach and other digestive disease as the outcome variable. Chronic digestive diseases include a wide spectrum of disorders, such as functional gastrointestinal disorders, inflammatory bowel disease, gastro-oesophageal reflux disease, and peptic ulcer disease, which have significant effects on the quality of life among older people. The risk factors of such chronic illnesses encompass sustained stressful life events (Mayer, 2000), the Helicobacter pylori infection which is likely due to lower socioeconomic status, poor sanitation, overpopulation and lack of safe drinking water, even during earlier life stages (Nurgalieva et al., 2002), and health risk behaviours such as smoking and excessive alcohol intake (Rosenstock, Jørgensen, Bonnevie & Andersen, 2003).

## 2 Methods

### 2.1 Participants and setting

The data in this study are from the China Health and Retirement Longitudinal Study (CHARLS) conducted by the National School of Development at Peking University. The CHARLS survey was carried out nationwide in 2011–2012, covering 28 out of 31 provinces, 150 districts/counties and 450 communities/villages, following a stratified (by per capita GDP of urban districts and rural counties) multi-stage (county/district-village/community-household) probability proportional to size (PPS) random sampling method (Zhao, Strauss & Yang, 2013). One person per household aged 45 years or older was selected, and they and their spouses were interviewed face-to-face by the trained interviewers using structured questionnaires. Respondents were asked to provide detailed information about themselves and their household members. In order to address the question of the extent to which adult children's migration in China affects the health of their elderly parents, the sample of interest was narrowed to those aged 60 or above who had at least one child.

The total survey respondents are 17,707, of whom 7358 are aged 60 and above. Those who have no children (N = 706) were excluded from the study sample. Another 157 respondents were excluded from the analysis because of missing values (not mutually exclusive) on chronic stomach and other digestive diseases (N = 17), marital status (N = 7), education (N = 11), income (N = 64), household wealth index (N = 91), smoking (N = 32), drinking (N = 34). The final analytical sample was 6495.

Signed informed consent was obtained from all participants by the CHARLS survey team before the data was collected (Zhao et al. 2013). Ethical approval for using the secondary data for this study has been obtained from the Ethics Committee in the University of Southampton (Ethics ID: 21228, 13/06/2016).

### 2.2 Measurements

#### 2.2.1 Chronic stomach and other digestive diseases

The central survey question analysed asked: ‘Have you been diagnosed with chronic conditions listed below by a doctor?’, and included two response options (yes or no). Stomach disease or other digestive diseases (except for tumours or cancer) was one of the 14 listed chronic morbidities/conditions. Other diseases included hypertension, dyslipidemia and diabetes or high blood sugar. In this study, the key outcome variable was dichotomous, distinguishing between respondents who had ever been diagnosed with a chronic stomach or other digestive disease, and those who had never been diagnosed with such disease.

#### 2.2.2 Migrant child

The survey enquired about the place of residence of each adult child not residing with the respondent, with seven response options (this household, but economically independent; the same or adjacent dwelling/courtyard with me; another household in this village/neighbourhood; another village/neighbourhood in this county/city; another county/city in this province; another province; abroad). The survey also collected information about the sex and birth date of each child. Having a migrant child was defined here as having any son aged 18 and above, and currently living in another county/city in the same province, or in another province. The age of eighteen was defined as the adult age by the Law of the People’s Republic of China on the Protection of Minors. A cross-county boundary was used to define internal migration as in the Chinese censuses (Duan & Sun, 2006). In the Chinese context, dependency in later life is mainly related to being dependent on one’s sons, as daughters frequently live elsewhere after their marriage and are expected to support their husband’s parents (Chou, 2011). Although married daughters play an increasing role in old age support, the patrilocal marriage practice affords sons much greater symbolic value (Liu, 2014). Thus the analysis focused on whether individuals have at least one migrant son as the key independent variable, which has three categories: No - Son, but do not have a migrant son; Yes - Have at least one migrant son; and Do not have a son.

#### 2.2.3 Other control variables

Covariates included the respondents’ age and sex; socio-economic factors including education, income and household wealth quintile; living arrangements; whether at least one child was living with the respondent or in the same county/city; health-risk behaviours such as smoking and alcohol drinking; and geographic factors such as rural or urban residence, and region. The household wealth quintile was computed using Principle Component Analysis based on 20 assets and housing characteristics.

### 2.3 Statistical analyses

We first explored the univariate associations of chronic digestive conditions with exposures and potential risk factors using the ᵡ^2^ test. In order to control for potential confounders, binary logistic regression was then used to evaluate the relationship between having migrant (adult) children and older parents’ chronic disease. The first model estimated the bivariate association between having migrant children and reporting a chronic disease. The second model examined this association after controlling for a range of covariates. All analyses were performed using SPSS version 22.0.

## 3 Results

### 3.1 Descriptive findings

Table 1 presents the descriptive statistics for the total analytical sample, i.e. 6495 individuals (3221 men and 3274 women) aged 60 and above with at least one child in China. The mean age of the sample was 68.4; the majority had no schooling or less than primary school education (81 percent) and lived in rural areas (77 percent). On average, older respondents had more than 3 children, and more than 1 son. The overall prevalence of chronic stomach or other digestive diseases was 22.1 percent. About 21.2 percent of older adults had at least one son living in another county or province in China. Among this sub-group, the prevalence of chronic disease was higher than the average, at 27 percent.

**Table 1:** Distribution of chronic stomach and other digestive diseases (unweighted).

**Variables**	**Frequency**	**Cases**	**% of digestive disease**	**P value (Pearson Chi square test)**
**Total**		**6495**	**22.1**	
***Migrant son***				
No	57.7	3745	20.8	0.000
Yes	21.2	1376	27.0	
Do not have a son	21.2	1374	20.6	
***Age group***				
60–69	62.3	4045	23.6	0.001
70–79	29.5	1918	20.0	
80+	8.2	532	18.2	
***Sex***				
Men	49.6	3221	20.5	0.003
Women	50.4	3274	23.6	
***Marital status***				
Currently married	78.7	5110	22.8	0.005
Widowed	21.3	1385	19.3	
***Education***				
No education	37.7	2448	22.8	0.000
Less than primary	43.6	2830	23.5	
Secondary and above	18.7	1217	17.4	
***Income***				
No wage or pension income	57.7	3750	23.8	0.000
Wage or pension	42.3	2745	19.8	
***Household wealth index***				
Lowest quintile	25.7	1669	24.7	0.000
Second	22.0	1427	24.4	
Middle	19.3	1253	20.8	
Fourth	18.3	1186	20.3	
Highest quintile	14.8	960	17.8	
***Smoking***				
Never	57.7	3746	22.3	0.674
Ever	42.3	2749	21.8	
***Drinking alcohol***				
Never	70.4	4570	22.9	0.018
Ever	29.6	1925	20.2	
***Number of children***				
1	13.7	889	19.9	0.134
2	23.4	1517	21.0	
3	25.9	1680	22.4	
4+	37.0	2409	23.3	
***At least one adult child living with or in the same county/city***				
No	11.0	712	23.9	0.220
Yes	89.0	5783	21.9	
***Living arrangement***				
Living alone	10.7	698	20.1	0.173
Living with spouse or others	89.3	5797	22.3	
***Residence***				
Rural	77.3	5022	23.7	0.000
Urban	22.7	1473	16.5	
***Region***				
West	32.2	2091	24.9	0.000
East	33.6	2185	17.8	
Central	34.2	2219	23.6	

The indicators of socio-economic status appear to have negative association with the report of chronic stomach or other digestive diseases. For instance, older people living in households in the highest wealth quintile presented a prevalence rate of 17.8 percent compared to 24.7 percent amongst those in the lowest wealth quintile. Older people without a wage or pension income had a higher prevalence of the disease than those with such income (23.8 compared to 19.8 percent), while those with a higher education showed a lower prevalence rate than those with lower education.

The prevalence of chronic stomach or other digestive diseases was higher among older women (23.6 percent) than older men (20.5 percent). Place of residence seems to play an important role, with elders living in rural areas being more likely to report a chronic disease than their urban counterparts (23.7 compared to 16.5 percent). There were also considerable regional variations in the prevalence of the disease, with a higher prevalence among older adults in the Western and Central regions compared to those in the Eastern region.

### 3.2 Multivariate analysis results

Table 2 presents the odds ratios of reporting a chronic stomach or other digestive diseases among older people. Model 1 shows the bi-variate relationship between having a migrant child and the occurrence of a chronic disease. The odds ratio of 1.40 suggests that older adults who have at least one son living in another county or province are more likely to report a chronic stomach or other digestive disease than those with children living closer. This effect was attenuated, but remains statistically significant, once other control variables were added (Model 2). The odds ratio of reporting the disease was higher among older adults with at least one adult son living in another county or province than among those with all sons living closer (OR = 1.29, 95% CI = 1.10–1.51). Interestingly, there was no difference between those with no son and no migrant son.

**Table 2:** Odds ratio of reporting chronic stomach and other digestive diseases.

**Variables**	**Model 1**		**Model 2**	
	**OR**	**95% CI**	**OR**	**95% CI**
***Migrant son*** No (ref)				
Yes	1.40***	(1.22–1.62)	1.29***	(1.10–1.51)
Do not have a son	0.99	(0.85–1.15)	0.99	(0.84–1.16)
***Age group*** 60–69 (ref)				
70–79			0.82**	(0.71–0.95)
80+			0.79	(0.61–1.02)
***Sex*** Men (ref)				
Women			1.30**	(1.10–1.54)
***Marital status*** Currently married (ref)				
Widowed			0.77**	(0.64–0.93)
***Education*** None (ref)				
Less than primary			1.09	(0.94–1.26)
Secondary and above			0.88	(0.72–1.09)
***Income*** No income (ref)				
Wage or pension			0.94	(0.82–1.07)
***Household wealth index*** Lowest quintile (ref)				
Second			1.01	(0.85–1.19)
Middle			0.87	(0.72–1.04)
Fourth			0.96	(0.79–1.17)
Highest quintile			1.02	(0.79–1.30)
***Smoking*** Never (ref)				
Ever			1.18*	(1.01–1.39)
***Drinking alcohol*** Never (ref)				
Ever			0.85*	(0.74–0.99)
***Number of children***			1.03	(0.98–1.08)
***At lease one adult child living with or in the same county/city No (ref)***				
***Yes***			0.99	(0.81–1.22)
***Living arrangement*** Living alone (ref)				
Living with spouse or others			0.94	(0.74–1.19)
***Residence*** Rural (ref)				
Urban			0.70***	(0.58–0.85)
***Region*** West (ref)				
East			0.68***	(0.58–0.79)
Centre			0.94	(0.82–1.09)
Nagelkerke R Square			0.006	0.032

Notes: OR = Odds Ratio; CI = Confidence Interval.

*** p < 0.001.

** p < 0.01.

* p < 0.05.

Among other important factors, age and gender play a role in the risk of reporting a chronic digestive disease. Women have higher odds than men; and individuals aged between 70–79 have lower odds than other age groups. Older people in rural areas show higher odds of a chronic stomach or other digestive disease than those living in urban areas. A significant regional variation was identified. Older people in the Eastern region show lower odds of reporting a chronic disease than those in the Western regions. Interestingly, health risk behaviour such as smoking and drinking alcohol have contrasting effects, with those who ever smoked being positively associated, and those who ever drink being negatively associated with the outcome. Living with others however was not statistically significantly different from living alone or with one’s spouse only. Education, household assets and income were not significantly associated with having a chronic digestive disease.

Nagelkerke R Square is 0.006 and 0.032 for Model 1 and Model 2 respectively. The second model fits the data significantly better than the first one (Chi-square = 115.06, P < 0.001).

## 4 Discussion

This study found that out-migration by an adult son is negatively associated with the health of parents ‘left behind’ in China. The prevalence of any of the diagnosed conditions of chronic stomach or other digestive diseases was higher among older people with a migrant son than among those without. More specifically, the odds ratio of reporting a disease was higher among older adults with at least one adult son living in another county or province than among those with their sons living closer, even after controlling for demographic, socio-economic and health behaviour factors. One explanation for this association may relate to existing research which shows that parents of migrant children may suffer from chronic stress, and may experience a change in their lifestyle towards less healthy behaviours, and a lower likelihood of using health care (Antman, 2010, Cai et al., 2012). Several qualitative studies have found that in transitional societies, out-migration of adult children is often accompanied by increased loneliness and isolation among their older parents, which are difficult to address through mechanisms of formal care (Miltiades, 2002, Vullnetari and King, 2008, Grant et al., 2009). Feelings of loneliness and isolation may in turn lead to constant worrying, anxiety, pessimism and depression among older parents, which can compromise physical health in the long run. In countries with strong son-preference such as China, India and Pakistan, living with, or nearby to son(s), has traditionally been a major source of old age security, whilst daughters who ‘marry’ out are seen as primarily providing support for their (new) parents-in-law. Such patriarchal support systems have existed for thousands of years in China and still persist especially in rural areas. Male children are considered as a source of strength for the family and sons are, at least culturally, still identified with as the ‘ideal’ source of support for parents in old age. In the absence of their migrant sons, the strong desire of being close to them is likely to lead to feelings of sadness, isolation, depression and loneliness in parents. Although the reduction in family sizes and increasing in internal or international migration has increased the need to rely on daughters as well as sons for old age support, it has been argued that such negative emotions are not be fully compensated by the daughters’ taking over care responsibilities which were traditionally expected of their brothers and sisters-in-law (Liu, 2014, Ashfaq et al., 2016, Gruijters, 2017). This is consistent with previous research findings in India where having a migrant son was negatively associated with older parents’ physical health (Falkingham et al., 2017).

Studies have suggested that chronic exposure to stress may result in a higher risk of disorders in one’s gastrointestinal function. Although biological, psychological and social factors contribute to the development of a digestive disease, numerous studies have suggested that digestive disorders such as functional gastrointestinal disorders, inflammatory bowel disease, gastro-oesophageal reflux disease and peptic ulcer disease, are particularly influenced by stress through the “brain-gut axis”, which refers to bidirectional signalling between the gastrointestinal tract and the brain regulated at neural, hormonal, and immunological levels (Mayer, 2000, Grenham et al., 2011, Hollander, 2003). In addition, individuals under stress might try to relieve their tension by consuming more unhealthy food, tobacco and alcohol which is harmful to one’s health (Epel, Lapidus, McEwen & Brownell, 2001).

Another explanation of this paper’s key finding relates to the issue of access to, and utilisation of, health care among older people. Previous research has found that older individuals with migrant children are less likely to receive intra-household social care, and to be taken to the hospital, compared to older people whose adult children live in closer proximity (Qin, 2008). The results are supportive of the hypothesis that migration of sons significantly increases the risk of chronic stomach and other digestive diseases among ‘left behind’ elderly parents in the contemporary Chinese context. The overall finding of a negative association between having a migrant child and parental health is consistent with previous studies in Mexico, Thailand, India and China (Guo et al., 2009, Antman, 2010, Falkingham et al., 2017, Ao et al., 2015).

This research also finds that gender, rural vs. urban and regional differences are independently associated with chronic disorders of the digestive system among older people. Older women are more likely to report such diseases than older men. This may be due to women having a relatively disadvantaged status, for instance being financially dependent on other family members (Cai et al., 2012) but being obliged to provide most of the care within the family (Liu, 2014), which might result in increased stress levels, with a negative effect on their health. Older people living in rural areas and those in the Western and Central regions have higher odds of reporting such diseases. This pattern of differences in the risk of chronic disease mirrors the pattern of regional socioeconomic development in China, with access to the state pension and health care systems favouring urban and East-region-based citizens, compared to rural citizens and other regions of the country (Du, 2013). These results reflect the fact that existing socioeconomic inequalities may influence older people's exposure and vulnerability to chronic diseases, which is in line with previous research highlighting that older people in rural areas and those living in the Western region have a lower disability-free life expectancy (Liu et al., 2010). A further possible reason for why older people in the eastern region display a lower odds of reporting a chronic disorder than those in the western areas may be related to differences in the eating habits between regions. Those dwelling in the western region are more likely to consume highly spiced ‘Sichaun’ cuisine, while those in eastern China eat lighter ‘Jiangsu’ or sweeter ‘Cantonese’ style meals. It is known that some spicy, fatty or fried foods are more likely to induce the gastroesophageal reflux disease (Surdea-Blaga, Negrutiu, Palage & Dumitrascu, 2017).

Interestingly, the results show that having ‘ever smoked’ is positively associated with the outcome, whilst having ‘ever drank’ is negatively associated with the outcome. This may reflect smokers’ increased susceptibility to Helicobacter pylori infection, which is believed to be associated with peptic ulcers (Bateson, 1993). The association between smoking and peptic ulcers is consistent with previous studies (Rosenstock et al., 2003). However, in the literature, the association between alcohol consumption and digestive disease, such as peptic ulcer is less conclusive. Epidemiological studies have found little evidence for the association between alcohol intake and peptic ulcer (e.g. Chou, 1994). Some research has, however, found that moderate alcohol, wine or beer intake may reduce the likelihood of ulcer disease, probably due to the beneficial effect of checking Helicobacter pylori infection (Rosenstock et al., 2003). The results from this study support this favourable effect.

Certain limitations of this study should be taken into consideration when interpreting the findings. Firstly, given that only the baseline survey data were analysed, it is not possible for this study to ascertain causality between having migrant adult children and the parents’ health outcomes in the origin community, allowing only for an identification of statistically significant associations between the two. However, a priori we might expect the parents of migrants to report better health outcomes than the parents of non-migrants, assuming the healthy selection of migrants and the parent-child health correlation due to genetic or environmental factors (Giles & Um, 2007), whilst in fact the converse is found. Secondly, the effect of children’s out-migration may simply be the outcome of earlier (but unobserved) processes which have led to the disease. That is, the reasons which brought about the children’s out-migration, for instance local income generation opportunity constraints, may also affect older parents’ risk of acquiring the disease. Future research would benefit from longitudinal data in order to investigate the extent to which older people’s disease occurs after their ‘exposure’ to the out-migration of their adult child(ren).

## 5 Conclusions

Despite its limitations, this study contributes to the current literature in several ways. Using a large and nationally representative sample of older adults, this study empirical evidence of a statistically significant relationship between children’s migration and poor parental health outcomes, which is a cause for concern. From a policy perspective, balancing economic growth and urbanisation with the maintenance and improvement in population health will be important in order to ameliorate any negative impact of such processes upon older people’s wellbeing. The uneven distribution of the prevalence of chronic digestive disease indicates that the disparities in the operation of the existing pension and medical care system between urban and rural areas, and between different regions of the country, must be narrowed.

## Conflict of interest statement

There is not conflict of interest.

## Declaration of sources of funding

This research was supported by the ESRC Centre for Population Change (grant number ES/K007394/1), with initial conceptualisation of the research supported by the AGEGlobe Network funded under the ESRC Ageing and Well-being in a Globalising World (grant number ES/K005979/1) and the ESRC Pathfinders grant (RES-238-25-0044) at the University of Southampton.

## Ethical statement

Signed informed consent was obtained from all participants by the CHARLS survey team before the data was collected (Zhao et al. 2013). Ethical approval for using the secondary data for this study has been obtained from the Ethics Committee in the University of Southampton (Ethics ID: 21228, 13/06/2016).
